# The immune synapse clears and excludes molecules above a size threshold

**DOI:** 10.1038/ncomms6479

**Published:** 2014-11-19

**Authors:** Adam N. R. Cartwright, Jeremy Griggs, Daniel M. Davis

**Affiliations:** 1 Manchester Collaborative Centre for Inflammation Research, University of Manchester, 46 Grafton Street, Manchester M13 9NT, UK; 2 Division of Cell and Molecular Biology, Imperial College London, London SW7 2AZ, UK; 3 GlaxoSmithKline, Gunnels Wood Road, Stevenage, Herts SG1 2NY, UK

## Abstract

Natural killer cells assess target cell health via interactions at the immune synapse (IS) that facilitates signal integration and directed secretion. Here we test whether the IS also functions as a gasket. Quantitative fluorescence microscopy of nanometer-scale dextrans within synapses formed by various effector-target cell conjugates reveal that molecules are excluded in a size-dependent manner at activating synapses. Dextran sized ≤4 nm move in and out of the IS, but access is significantly reduced (by >50%) for dextran sized 10–13 nm, and dextran ≥32 nm is almost entirely excluded. Depolymerization of F-actin abrogated exclusion. Unexpectedly, larger-sized dextrans are cleared as the IS assembles in a zipper-like manner. Monoclonal antibodies are also excluded from the IS but smaller single-domain antibodies are able to penetrate. Therefore, the IS can clear and exclude molecules above a size threshold, and drugs designed to target synaptic cytokines or cytotoxic proteins must fit these dimensions.

## Abstract (web summary)

Natural killer cells can be switched on or off by the immune synapse formed with another cell. Here, the authors show that when a natural killer cell is activated, the immune synapse also functions to clear and exclude extracellular molecules, including antibodies, in a size-dependent manner.

## Graphical abstract

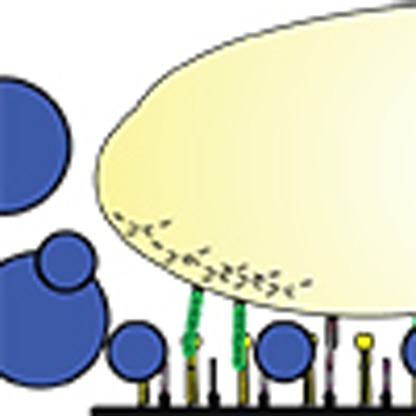

Natural killer (NK) cells are large granular lymphocytes that aid immune responses through cytokine secretion and direct lysis of infected or transformed cells^1^,^2^. These effector functions can be triggered by transient contacts that NK cells make with other cells, that is, at the NK cell immune synapse (IS)^3^,^4^. If a dominant activating signal is received (for example, via CD16 or NK group 2 member D), a cytolytic response may be triggered in which cytotoxic mediators are secreted across the synapse^5^. Upon encountering a healthy cell, signalling from inhibitory receptor–ligand interactions dominate the outcome of the interaction (for example, via killer immunoglobulin-like receptors (KIR))^6^, resulting in a much shorter-lived synapse and no release of cytolytic proteins^7^,^8^.

A relatively unexplored function of the IS is the potential for forming a gasket, or seal, around the synapse. A previous study has shown that monoclonal antibodies (mAbs) against perforin were unable to block the action of this protein^9^. The reason for this may be that a gasket is formed by a dense accumulation of activating and adhesion receptor–ligand complexes and/or ruffling of the cell membrane, which could potentially restrict access of extracellular molecules into the synapse. Here, we establish that the synapse does not completely seal the synaptic cleft, but rather excludes extracellular molecules in a size-dependent manner. An intact F-actin structure at the cell–cell interface is necessary for this size-dependent exclusion. Unexpectedly, we also found that larger molecules are cleared from the IS during its formation. In addition, we report that while IgG antibodies are excluded from the synapse, smaller single-domain antibodies (dAbs) are able to access the synaptic cleft. These data establish that the size threshold should be taken into account in the design of antibody-based therapies that target cytokines or cytolytic proteins secreted across ISs.

## Results

### Size-dependent exclusion from the IS

**Figure 1:**
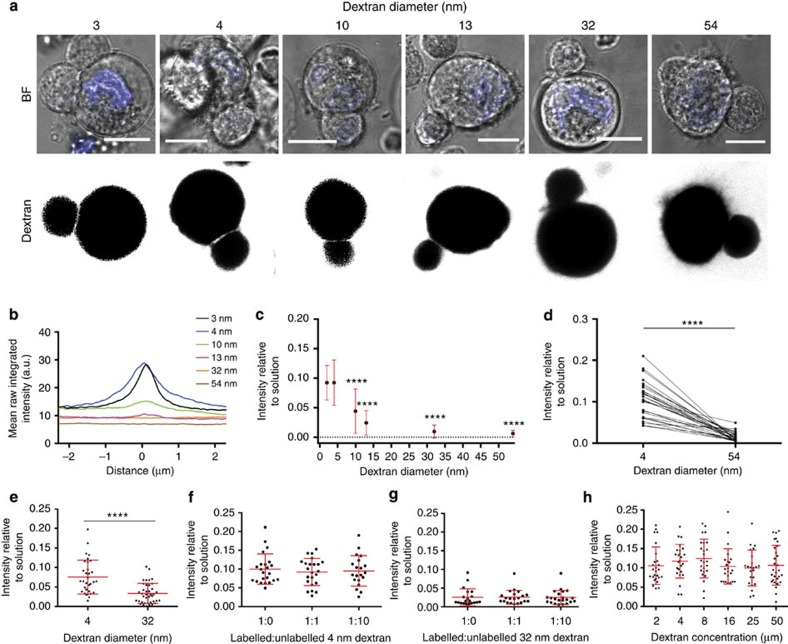
Dextran is excluded from the activating pNK cell synapse in a size-dependent manner. (**a**) Panels show bright-field (BF) images of pNK cells and 221 target cells overlaid with target cell nuclear staining (upper row) and the corresponding fluorescence image of dextran (lower row), size indicated. Scale bars, 10 μm. (**b**) Graph shows the mean raw fluorescence intensity of various sized dextrans (as indicated) for a line drawn perpendicular to the synapse. (**c**) Graph shows the relative intensity of dextran for each size tested. Error bars represent mean±s.d. *n*=38, 32, 33, 31, 38 and 35 conjugates from three independent experiments. Data were analysed using a one-way analysis of variance (ANOVA) with Tukey corrections. ****P<0.0001. (**d**) Graph shows the relative intensities of 4 and 54 nm dextran within the same synapse (connecting lines). *n*=29 conjugates from three independent experiments. Data were analysed using a Student’s *t*-test with Welch’s corrections. ****P<0.0001. (**e**) Graph shows the relative intensities of undiluted 4nm dextran or 32 nm dextran mixed 1:10 fluorescein-labelled:unlabelled within synapses formed by YTS cells and 221 cells. Error bars represent mean±s.d. *n*=32 conjugates for 4 nm dextran and 39 conjugates for 32 nm dextran from three independent experiments. Data were analysed using a two-tailed Student’s *t*-test with Welch’s corrections. ****P<0.0001. (**f**,**g**) Graphs show the relative intensities of 4 and 32 nm dextran within synapses formed by YTS cells and 221 cells for each labelled:unlabelled mixture ratio, as indicated. Error bars represent mean±s.d. *n*=23, 23 and 20 conjugates for 4 nm dextran and 22, 21 and 22 conjugates for 32 nm dextran from three independent experiments. Data were analysed using a one-way ANOVA with Tukey corrections. (**h**) Graph shows the relative intensities of 4 nm dextran at different concentrations, as indicated, within synapses formed by YTS cells and 221 cells. Error bars represent mean±s.d. *n*=26, 25, 25, 23, 23 and 29 conjugates from three independent experiments. Data were analysed using a one-way ANOVA with Tukey corrections.

To test whether there was a size-dependent requirement for molecules to enter the NK cell IS, fluorescein-labelled dextrans of varying molecular weight, 3–2,000 kDa, previously measured to have hydrodynamic diameters 3–54 nm (refs 10, 11), were added to primary human NK (pNK) cells or the NK cell line YTS (a well-characterised subclone of the YT cell line) co-incubated with 721.221 target cells (221 hereon). 221 cells are Epstein-Barr virus (EBV)-transformed B cells that are well established sensitive targets for both pNK cells and the cell line YTS, through a lack of expression of endogenous class I major histocompatibility complex (MHC) proteins^12^. Conjugates were imaged by confocal microscopy that revealed that as dextran size increased, penetration into synapses decreased in those formed by both pNK cells (Fig. 1a) and YTS cells (Supplementary Fig. 1a)

To quantify the access of differently sized dextrans into the IS, the fluorescence intensity of dextran in the solution, cell body and synapse was measured along a line perpendicular to the IS (Supplementary Figs 2 and 3 for pNK cells and YTS cells, respectively). The peak in fluorescence intensity within the synapse over the background intensity within the cell body indicates the presence of dextran. Despite the synaptic cleft spanning ~25 nm (ref. 13), the width of fluorescence peaks are ~1–2 μm owing to the diffraction that makes the point-spread function (PSF) of the microscope larger than the cleft size. Importantly, however, as dextran size increased, the peak of intensity at the synapse decreased, indicating less dextran being present in the synapse (Fig. 1b and Supplementary Fig. 1b for pNK cells and YTS cells, respectively).

To compare measurements across different experiments, the dextran fluorescence intensity was measured at the synapse in comparison with its intensity in the bulk extracellular solution (Fig. 1c and Supplementary Fig. 1c for pNK cells and YTS cells, respectively). The relative intensities of 3- and 4-nm-sized dextrans in synapses formed by either pNK cells (0.09±0.03 and 0.09±0.04, respectively) or YTS cells (0.09±0.04 and 0.09±0.05, respectively) were comparable. The reason this value is <1 is because the PSF of the microscope will capture a larger volume of fluorescence in the solution compared with the fluorescence from the narrow synaptic cleft. Importantly, however, as dextran size increased, the penetration of the synapse decreased significantly. The intensity of 10 nm dextran decreased by 50% and 44% compared with 3 or 4 nm dextran, in synapses formed by either pNK cells or YTS cells, respectively. The 32-nm (0.01±0.01 and 0.03±0.02) and the 54-nm (relative intensity 0.01±0.01 for both) dextrans were almost entirely excluded from synapses formed by pNK cells or YTS cells, respectively.

To test whether dextrans of different size would enter the exact same synapses to a different extent, equal concentrations (2.5 μM) of dextrans with diameters 4 and 54 nm, labelled with Texas red or fluorescein, respectively, were added together to pNK cells or YTS cells co-incubated with 221 target cells. Confocal microscopy revealed that the 4-nm dextran was present within synapses formed by pNK cells and YTS cells (0.11±0.04 and 0.14±0.06, respectively), whereas the 54-nm dextran was excluded (0.01±0.01 for both) from the same synapses (Fig. 1d and Supplementary Fig. 1d for pNK cells and YTS cells, respectively). Confirming that the dyes themselves were not important here (as, for example, they result in dextran having different charges), 4-nm-sized dextran labelled with either Texas Red (neutral charge) or Alexa Fluor 647 (negative charge) could enter the synapse equally well (Supplementary Fig. 4). Together, these data indicate that small molecules were able to access the IS, whereas larger molecules were excluded.

A caveat to these experiments is that each size of dextran has a different number of fluorophores and, thus, microscope settings were adjusted in each case. In order to test that this did not introduce artefacts, a fluorescein-labelled 32-nm dextran was mixed with an unlabelled 32-nm dextran to dilute its fluorescence by a 1:10 ratio and added to YTS cells co-incubated with 221 cells. The same microscope settings could then be used to image both the 4- and 32-nm dextrans (both at 2.5 μM). Here, the 32-nm dextran, but not the 4-nm dextran, was largely excluded from the IS (relative intensities being 0.03±0.03 and 0.08±0.04, respectively; Fig. 1e). We also tested whether or not the concentration of the fluorophore was a factor in measuring the extent to which dextran penetrated the synapse by comparing 4 or 32 nm fluorescein-labelled and unlabelled dextrans mixed at different ratios and imaged with the same microscope settings. We found that neither a 1- or 10-fold dilution of fluorescence affected the extent to which dextran was detected in the synapse compared with undiluted fluorescent dextran (Fig. 1f,g for 4 or 32 nm dextran, respectively). Finally, we tested whether the concentration of dextran had any effect on penetration of the synapse. Using fluorescein-labelled 4 nm dextran at a range of concentrations, the fluorescence intensity within the synapse relative to the solution remained the same (Fig. 1h), demonstrating that concentration had no aberrant effect on our measurement of size exclusion or penetration into the synapse. Together, these data establish that neither the concentration of dextran nor its extent of labelling affected its ability to penetrate the synapse—rather, physical size determines the extent to which dextran can fill the IS.

### Size-dependent exclusion with different target cells

**Figure 2:**
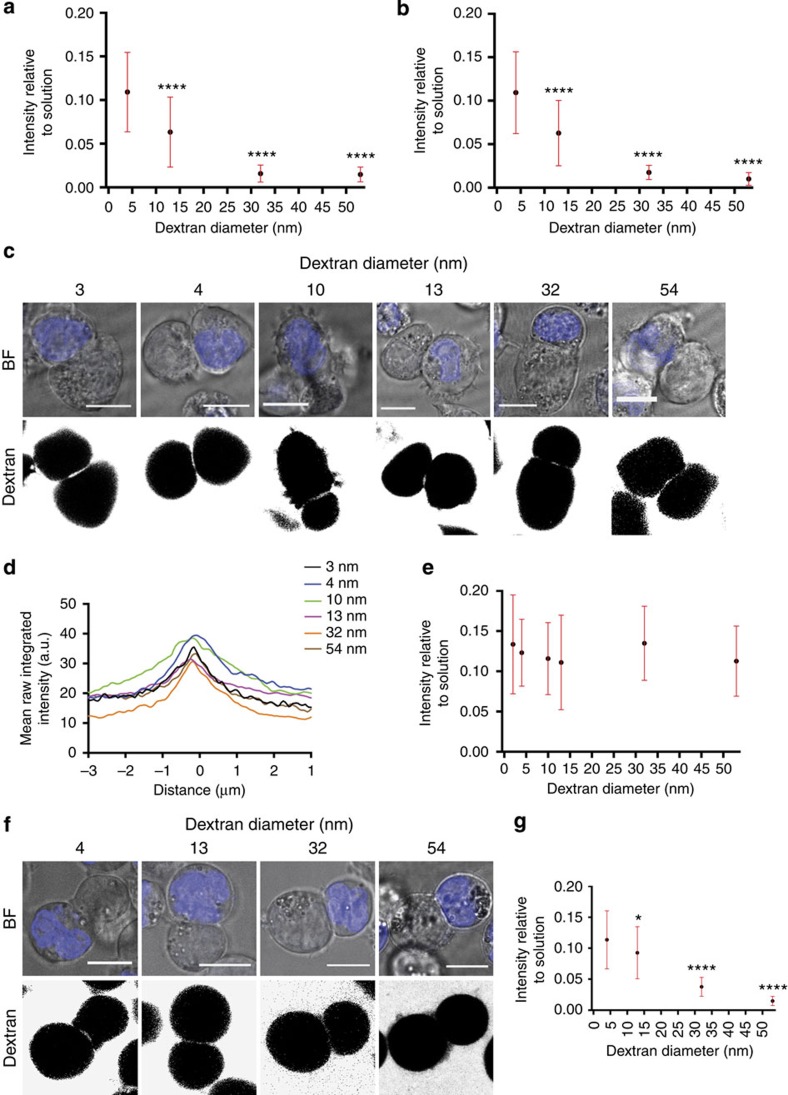
Dextran is excluded in a size-dependent manner by activating but not inhibitory synapses. (**a**,**b**) Graphs show the mean relative fluorescence intensity of dextran of the sizes indicated for synapses formed by pNK cells co-incubated with (**a**) Daudi cells or (**b**) K562 cells. Error bars show mean±s.d. of all data points. *n*=40, 39, 35 and 35 conjugates for Daudi cells and 40, 38, 38 and 42 conjugates for K562 cells from three independent experiments. Data were analysed using a one-way analysis of variance (ANOVA) with Tukey corrections. ****P<0.0001. (**c**) Panels show bright-field (BF) images of YTS/KIR2DL1 cells in conjugate with 221/Cw6 target cells overlaid with nuclear stained target cell (blue;upper row) and the corresponding fluorescence image of dextrans (lower row), sizes as indicated. Scale bars, 10 μm. (**d**) Graphs show mean raw fluorescence intensities of dextran (at the size indicated) along a line drawn perpendicular to the synapse. (**e**) Graph shows the mean relative fluorescence intensities of dextran within synapses formed by YTS/KIR2DL1 and 221/Cw6 cells. Bars show the mean for all data points. *n*=42, 45, 41, 45, 35 and 35 from three independent experiments. Data were analysed using a one-way ANOVA with Tukey corrections. (**f**) Panels show BF images overlaid with nuclear stained target cell (upper row) and the corresponding fluorescence image of dextran (lower row), size as indicated, of Jurkat cells in conjugate with superantigen-loaded Raji target cells. Scale bars, 10 μm. (**g**) Graph shows the mean relative fluorescence intensities of each dextran within synapses formed by T cells and Raji target cells. Bars show mean from all data points. *n*=34, 36, 33 and 37 from three independent experiments. Data were analysed using a one-way ANOVA with Tukey corrections. *P<0.1, ****P<0.0001.

We next tested whether synapses formed between NK cells and different target cells similarly excluded extracellular molecules in a size-dependent manner. To test this, pNK cells were co-incubated with a B-cell lymphoblast cell line, Daudi, or a myeloid leukaemia cell line, K562, and differently sized fluorescein-labelled dextrans. As observed with 221 target cells, the IS formed between pNK cells and Daudi (Fig. 2a) or K562 (Fig. 2b) cells excluded the 32-nm (relative intensity 0.02±0.01 with both targets) and the 54-nm (0.02±0.01 and 0.01±0.01, respectively) dextrans, while the 4-nm (0.11±0.05 with both targets) and the 13-nm (0.06±0.04 with both targets) dextrans were able to access the synapse. The intensities of dextran within synapses were comparable to those measured when pNK cells were co-incubated with 221 cells (Fig. 1). These data establish that, regardless of the target cell, NK cells exclude extracellular molecules above a size threshold from the IS.

### The inhibitory synapse does not exclude dextran

An inhibitory synapse is a short-lived, yet structured, interface between NK cells and healthy cells, in which signals from inhibitory receptors dominate the outcome of the interaction and prevent the release of cytolytic mediators^14^. To test whether or not inhibitory NK cell synapses exclude extracellular molecules, we imaged YTS cells transfected to express the inhibitory NK cell receptor KIR2DL1 (YTS/KIR2DL1) co-incubated with 221 cells transfected to express an inhibitory ligand for KIR2DL1, the class I MHC protein HLA-Cw6 (221/Cw6) (ref. 15) and dextrans of various sizes (Fig. 2c). The fluorescence intensity of dextran was measured across the solution, cell bodies and synapse, in the same manner as activating synapses (Supplementary Fig. 5). In contrast to the activating synapse formed by YTS and 221 cells, a peak of fluorescence intensity within the synapse was detected for dextran of all sizes (Fig. 2d). Moreover, the relative fluorescence intensities within the inhibitory synapse were similar, not varying significantly, for all dextran sizes used (0.11±0.06–0.14±0.04; Fig. 2e). This establishes that the inhibitory synapse does not exclude extracellular molecules in a size-dependent manner.

### Size-dependent exclusion from the T-cell synapse

The best studied IS is that which is formed between T cells and antigen-presenting cells^16^. The dimensions of many of the key receptor–ligand complexes at T-cell synapses are similar to those at the NK cell IS^17^,^18^. To test whether or not the IS formed by T cells also excludes extracellular molecules in a size-dependent manner, 4-, 13-, 32- and 54-nm-sized fluorescein-labelled dextrans were added to the T-cell leukaemic cell line Jurkat, co-incubated with a Burkitt lymphoma cell line, Raji, that had been loaded with the superantigen Enterotoxin E. Conjugates were imaged by confocal microscopy (Fig. 2f). The 4- and 13-nm dextran were able to penetrate synapses (relative fluorescence 0.11±0.05 and 0.09±0.04, respectively). However, the 32- and 54-nm dextrans were largely excluded from the synapse formed by Jurkat T cells (0.04±0.02 and 0.01±0.01, respectively; Fig. 2g). This shows that the T-cell IS excludes extracellular molecules in a size-dependent manner, similar to the activating synapse formed by NK cells.

### Dextran moves freely in and out of the synapse

**Figure 3:**
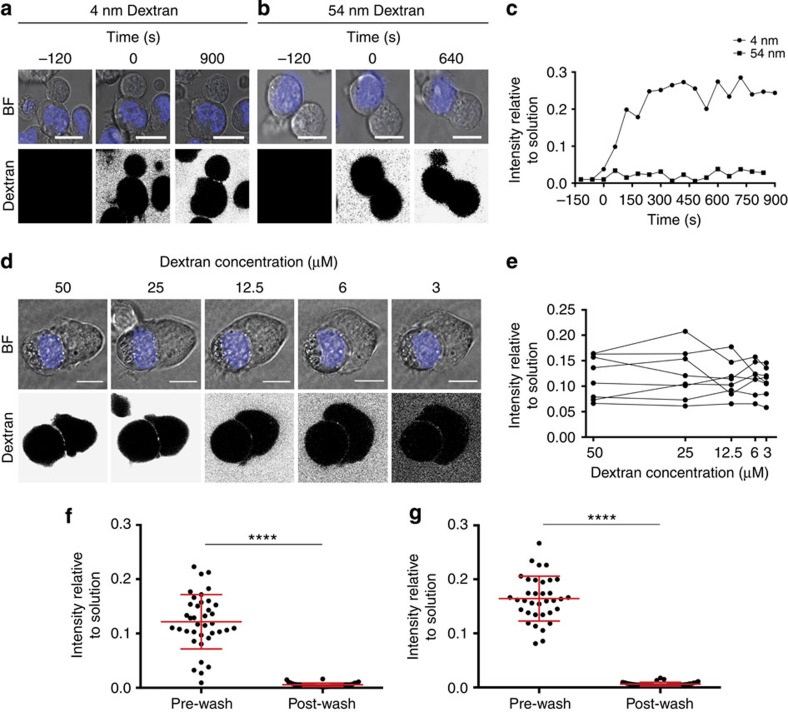
Dextran below the size exclusion threshold can move in and out of the IS. (**a**,**b**) Representative time-lapse confocal images of YTS and 221 cells before, and following, the addition of (**a**) 4 nm dextran or (**b**) 54 nm dextran (added at time=0). Panels show bright-field (BF) images overlaid with a nuclear stain marking target cells (blue) and corresponding fluorescence images of fluorescent dextran at the times were indicated. Scale bars, 10 μm. (**c**) Relative fluorescence intensity of 4 nm (·) and 54 nm (▪) dextran within the synapse at each time point, where time=0 indicates the time at which dextran was added. *n*=5 for each dextran size. (**d**) Representative images of a YTS cells co-incubated with 221 cells as the extracellular 4 nm dextran was diluted, final concentration as indicated. Panels show BF images overlaid with a nuclear stain marking target cells and corresponding fluorescence images of fluorescent dextran at each dilution. Scale bars, 10 μm. (**e**) Graph shows the mean relative fluorescence intensity of dextran within the synapse of the same conjugate as the extracellular dextran was diluted (connecting lines). *n*=8. Data were analysed using a one-way analysis of variance with Tukey corrections. (**f**,**g**) Graphs show relative fluorescence intensities of dextran in synapses formed by (**f**) YTS cells or (**g**) pNK cells co-incubated with 221 target cells, before (pre-wash) and following washing (post-wash). Fluorescence intensity of dextran in the synapse is shown relative to the mean intensity of the extracellular solution before washing. Error bars represent mean±s.d. *n*=38 and 40 for YTS cells and 35 and 39 for pNK cells from three independent experiments. Data were analysed using a two-tailed Student’s *t*-test with Welch’s corrections. ****P<0.0001.

Our initial experiments established that dextran could access the synapse, in a size-dependent manner, when present during the formation of a synapse. To test whether or not dextran can enter synapses that have already formed, YTS and 221 cell conjugates were co-incubated on poly-L-lysine-coated slides and imaged by time-lapse confocal microscopy. During imaging, 4- (Fig. 3a) or 54-nm (Fig. 3b) fluorescein-labelled dextran was added to the solution. The 4-nm dextran was detected in the synapse almost immediately following its addition, followed by an increase in fluorescence intensity over the first 2–3 min before reaching a plateau. In contrast, the 54-nm dextran was unable to penetrate the IS (Fig. 3c), consistent with our data showing that the IS excludes larger molecules (Fig. 1 and Supplementary Fig. 1). Therefore, small dextrans can enter the IS, even after its formation.

To test whether or not dextran could move out from the synapse into the extracellular solution, YTS and 221 cells were co-incubated on poly-L-lysine-coated slides in the presence of a 4-nm dextran and imaged by confocal microscopy as dextran was sequentially diluted with medium. The same cell–cell conjugate was imaged following each sequential dilution (Fig. 3d), and the fluorescence intensity of dextran in the synapse was measured relative to the extracellular solution (Fig. 3e). Following each dilution, the fluorescence intensity within the synapse relative to the solution did not vary significantly. This establishes that dextran within the synapse equilibrates with the extracellular solution, and that small-sized dextrans are able to move in and out of the IS.

In a separate experiment, we investigated the effect of complete removal of dextran from the extracellular solution. YTS cells or pNK cells were co-incubated with 221 target cells on poly-L-lysine-coated slides in the presence of fluorescein-labelled 4-nm dextran. Conjugates were then imaged by confocal microscopy to detect dextran within the synapses (pre-wash); the medium was then removed and replaced with dextran-free medium and conjugates were immediately imaged again by confocal microscopy (post-wash). The initial fluorescence intensities of dextran (relative to the solution) in synapses formed by YTS or pNK cells were 0.12±0.05 and 0.16±0.04, respectively. Following removal of extracellular dextran, fluorescence intensity (compared with the mean intensity of the solution before washing) reduced significantly to 0.006±0.003 for both YTS cells (Fig. 3f) and pNK cells (Fig. 3g). These data demonstrate that small-sized dextran is not trapped and can be washed out of the IS.

### Synapse formation clears larger extracellular molecules

**Figure 4:**
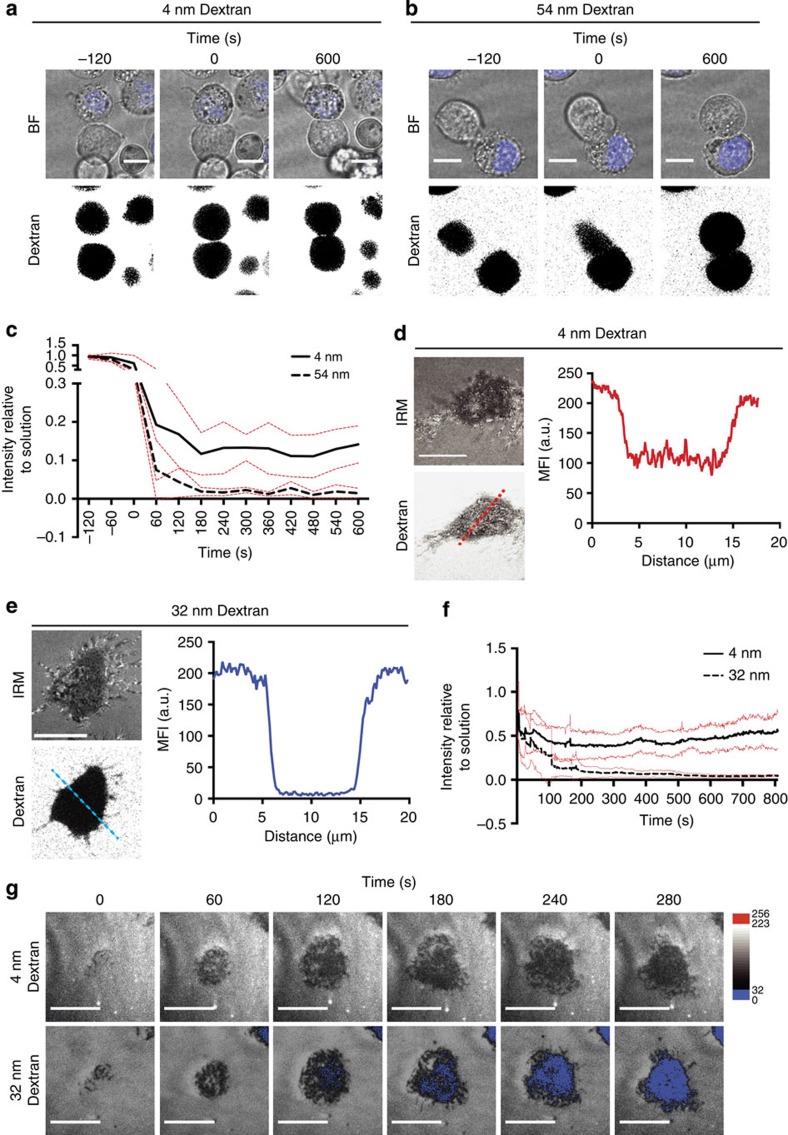
Larger extracellular molecules are cleared from the IS during assembly. (**a**,**b**) Panels show representative time-lapse bright-field (BF) images overlaid with target cell nuclear stain (upper row) and corresponding fluorescence image (lower row) at time points indicated of synapse formation between a YTS cell and a 221 cell in the presence of (**a**) 4 or (**b**) 54 nm dextran on fibronectin-coated slides. Time=0 indicates initial cell–cell contact. Scale bars, 10 μm. (**c**) Graph shows relative fluorescence intensity over time of 4 nm (−) and 32 nm (--) dextran within the synapses of YTS cells and 221 target cells before and during synapse formation. Time=0 indicates initial cell–cell contact. Red lines indicate 95% confidence interval (CI). *n*=5 for 4 nm dextran and *n*=5 for 32 nm dextran. (**d**,**e**) pNK cells were plated on slides coated with MICA-Fc and ICAM1-Fc in the presence of (**d**) 4 nm or (**e**) 32 nm dextran. Panels show interference reflection microscopy (IRM) image and corresponding fluorescence image of synapse formation after 500 s contact with the coated slides. Graphs show the mean fluorescence intensity (MFI) of 4 nm (red) and 32 nm (blue) dextran along the dotted lines indicated in fluorescence images. Scale bar, 10 μm. (**f**) Graph shows the mean relative intensity of dextran over time within the cell–slide interface during synapse assembly in the presence of 4 nm (−) and 32 nm (--) dextran. Red lines represent 95% CI. *n*=5 for 4 nm dextran and *n*=5 for 32 nm dextran. (**g**) pNK cells were plated with Texas Red-labelled 4 nm dextran and fluorescein-labelled 32 nm dextran on MICA-Fc- and ICAM1-Fc-coated slides. Cell–slide contact and synapse formation was then imaged by TIRF microscopy for 5 min, taking images every 20 s. Panels show representative images of initial contact (time=0) and synapse formation at the time points indicated. A HiLo threshold was used to identify pixel intensities below 32 a.u. (blue) and above 223 a.u. (red). Scale bars, 10 μm. Images are representative of six cells from five independent experiments.

The fact that larger-sized dextrans, present from the time of initial contact between two cells, appear to be excluded from synapses (Figs 1 and 2 and Supplementary Fig. 1) raises the question of what happens to dextran molecules caught in the synaptic cleft when the initial contact is made. To investigate this, we set out to image fluorescently labelled dextran from the moment of initial cell–cell contact and during synapse assembly. YTS and 221 cells were co-incubated on fibronectin-coated slides in the presence of 4 or 54-nm-sized dextrans, and synapse formation was imaged by time-lapse confocal microscopy (Fig. 4a for 4 nm dextran and Fig. 4b for 54 nm dextran). Both 4 and 54 nm dextrans were initially detected between the two cells before conjugation, followed by an initial decrease in the fluorescence intensity of both dextrans as the synapse formed. This is because the PSF of the microscope is larger than the narrow synaptic cleft. However, as the synapse assembled, the intensity of the 54-nm dextran reduced to a far greater extent than fluorescence from the 4-nm dextran (Fig. 4c). These data show that the formation and assembly of the synapse causes a size-dependent clearance of dextran.

To further investigate the clearance of dextran from the synapse, we used interference reflection microscopy together with confocal fluorescence microscopy to image dextran at the contact site between a pNK cell and an activating surface. Microscope slides were coated with MICA-Fc and ICAM1-Fc chimera proteins to stimulate NK cells, as described previously^19^. pNK cells were plated on the slides in medium containing fluorescein-labelled 4 or 32-nm-sized dextrans, and synapse formation was imaged (Fig. 4d for 4-nm dextran and Fig. 4e for 32-nm dextran). The region of close contact between the NK cell and the activating surface appears dark in the interference reflection microscopy image. Images showing NK cell synapse formation in the presence of 4 nm dextran demonstrate that dextran enters the synapse where the membrane is tight to the slide. Conversely, 32-nm-sized dextran is excluded, implying that the cell–slide contact excludes larger dextran. The fluorescence intensity of dextran at this point of close contact was measured relative to the intensity of dextran within the extracellular solution. Immediately following contact with the slide, the fluorescence intensity of dextran decreased, as the depth of the synaptic cleft will be narrower than the depth of imaging achieved in the solution away from the cell. However, as the NK cell spread on the slide, the fluorescence intensity of the larger 32 nm dextran, but not the 4-nm dextran, continued to decrease—until it was undetectable (Fig. 4f).

Time-lapse total internal reflection fluorescence (TIRF) microscopy of pNK cells interacting with slides coated with MICA-Fc and ICAM1-Fc was also performed in the presence of both 4 and 32 nm dextrans (Fig. 4g and Supplementary Movie 1, for 4 nm dextran, and Supplementary Movie 2 for 32 nm dextran). To identify a presence or lack of fluorescent dextran, a look-up table (HiLo) was used to identify pixels with intensities below (blue) or above (red) thresholds in the 8-bit images. As the NK cell spread on the slide, the images of 4 nm dextran show very few blue pixels, whereas for 32 nm dextran the cell–slide interface clearly turns blue, indicating a loss of the larger fluorescent dextran. We also quantitatively compared the area of close contact between the cell and slide (black pixels) versus the region where dextran was excluded over time (Supplementary Fig. 6). In some cases, dextran exclusion occurred at a slight delay following tight contact with the slide, but in other cases the exclusion of 32 nm dextran was concurrent with cell spreading within the time resolution used. This further establishes that larger dextran is squeezed out of the synapse as it spreads in a zipper-like fashion.

### Intact F-actin is required to exclude dextran

Hallmarks of an activating IS includes the assembly of a dense filamentous (F)-actin ring at the synapse periphery^4^,^20^,^21^. An actin mesh also underlies the central region of the synapse that supports protein complexes at the synapse^19^,^22^. To test whether or not the actin cytoskeleton influenced exclusion of the dextran from the synapse, we used 1 μM Latrunculin A (Lat A) or 0.5 μM Jasplakinolide (Jasp) to depolymerize^23^ or stabilize^24^ actin, respectively.

Confirming that these drugs, at the concentrations used, affected NK cell F-actin, Lat A depolymerized actin in pNK cells, YTS cells and 221 cells (Supplementary Fig. 7a–d) and Jasp significantly decreased pNK cell migration on fibronectin-coated slides (Supplementary Fig. 7e–h). After a 5-min incubation with either drug, cell viability was not affected (Supplementary Fig. 7i–k).

**Figure 5:**
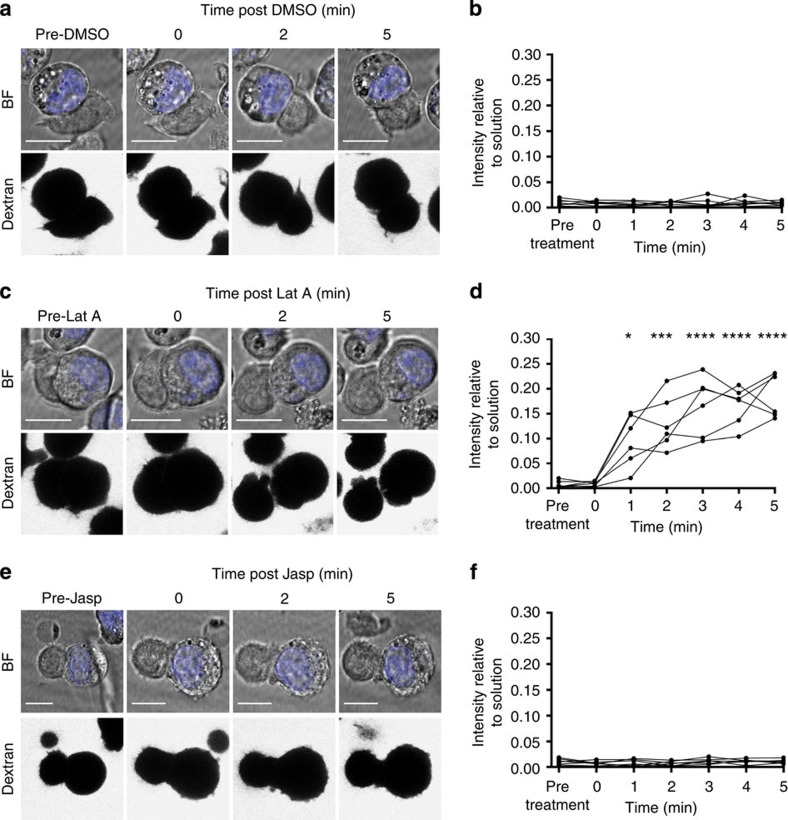
The actin cytoskeleton is important for the exclusion of extracellular molecules from the synapse. pNK cells were co-incubated with 221 target cells and fluorescein-labelled 32-nm-sized dextran. Conjugates were imaged before and following the addition of DMSO, 1 μM Lat A or 0.5 μM Jasp. Images show bright-field (BF) images overlaid with target cell nuclear staining and corresponding fluorescence images of 32 nm dextran before and following the addition of (**a**) DMSO, (**c**) 1 μM Lat A and (**e**) 0.5 μM Jasp. Scale bars, 10 μm. Graphs show the relative intensity of dextran within the same synapse at each time point (connecting lines) prior and after addition of (**b**) DMSO, (**d**) 1 μM Lat A and (**f**) 0.5 μM Jasp, as indicated. Data are from 7, 6 and 6 conjugates for DMSO, Lat A and Jasp, respectively. Data were analysed using a one-way analysis of variance with Tukey corrections. *P<0.1, ***P<0.001, ****P<0.0001.

To test whether F-actin was required for the exclusion of larger dextran, pNK cells or YTS cells in conjugates with 221 target cells were co-incubated with 32 nm dextran and imaged by confocal microscopy before addition of the drug, immediately after addition and once per minute afterwards. Following addition of the vehicle control, dimethylsulphoxide (DMSO), did not affect the ability of either pNK cells or YTS cells to exclude 32 nm dextran (mean relative intensity pretreatment to 5 min after DMSO addition was 0.01±0.01–0.009±0.004 for pNK cells and 0.03±0.01–0.02±0.01 for YTS cells; Fig. 5a,b for pNK cells and Supplementary Fig. 8a,b for YTS cells). However, following the addition of Lat A, the fluorescence intensity of 32 nm dextran within the synapse began to increase (Fig. 5c for pNK cells and Supplementary Fig. 8c for YTS cells). Compared with mean relative intensities within the synapse before Lat A addition, after 5 min the mean relative intensity of the 32-nm dextran had increased from 0.01±0.01 to 0.19±0.05 (Fig. 5d) and in YTS cell synapses from 0.02±0.02 to 0.26±0.11 (Supplementary Fig. 8d). In contrast, the addition of Jasp did not affect the ability of the IS to exclude 32 nm dextran (mean relative intensity pretreatment to 5 min after Jasp addition was 0.01±0.01–0.011±0.004 for pNK cells and 0.03±0.01–0.04±0.02 for YTS cells; Fig. 5e,f for pNK cell synapses and Supplementary Fig. 8e,f for YTS cell synapses). This suggests that the actin cytoskeletal scaffold that forms at the IS is necessary for the exclusion of dextran in a size-dependent manner, but that once the synapse has formed, active rearrangement of F-actin is not required.

### Access of proteins into the synapse also depends on their dimensions

**Figure 6:**
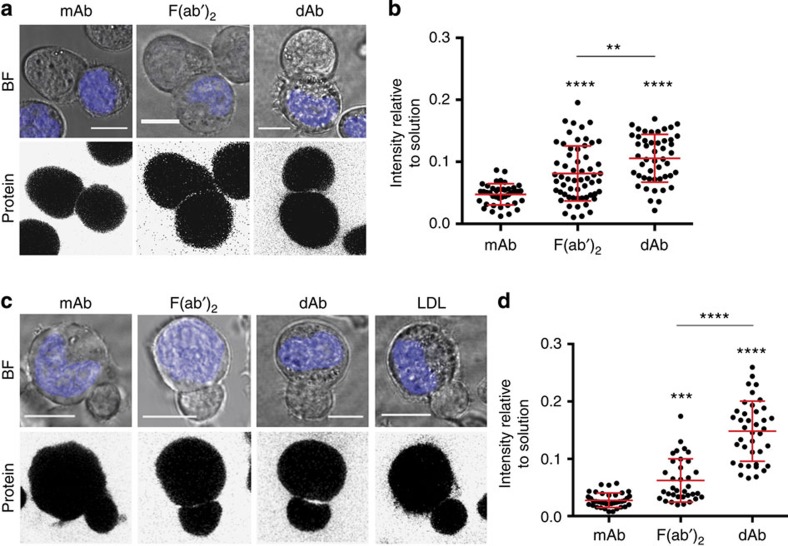
Proteins are excluded from the IS in a size-dependent manner. Representative images of (**a**) YTS and (**c**) pNK cells in contact with 221 target cells co-incubated with differently sized fluorescently labelled proteins as indicated. Panels show bright-field (BF) images overlaid with a nuclear stain marking target cells and corresponding fluorescence images of labelled proteins. Scale bars, 10 μm. (**b**) Graph shows the mean relative fluorescence intensities of each protein within synapses formed by YTS cells. Bars show mean of all data points. *n*=41, 59 and 49 from three independent experiments. Data were analysed using a one-way analysis of variance (ANOVA) with Tukey corrections. **P<0.01, ****P<0.0001. (**d**) Graph shows the mean relative fluorescence intensities of each protein within synapses formed by pNK cells. Bars show mean of all data points. *n*=38, 35, 39 and 54 from three independent experiments. Data were analysed using a one-way ANOVA with Tukey corrections. **P<0.01, ***P<0.001, ****P<0.0001.

Antibodies against perforin are unable to block NK cell cytotoxicity^9^. One explanation for this could be that antibodies cannot easily penetrate the synapse owing to the size restrictions identified here. To test this specifically, we investigated the ability of antibody-based proteins of different sizes to penetrate the IS. We compared fluorescently labelled mAbs, F(ab′)_2_ fragments and dAbs, whose longest dimensions have previously been measured as 16 (ref. 25), 9 (ref. 10) and 4 nm (ref. 26), respectively. YTS cells or pNK cells were co-incubated with 221 target cells in the presence of these antibodies and then were imaged by confocal microscopy (Fig. 6a,c, respectively).

In synapses formed by YTS cells (Fig. 6a), fluorescently labelled mAb could be detected to some extent (synapse intensity relative to the extracellular solution 0.05±0.02), F(ab′)_2_ fragments were more easily detected within synapses (0.08±0.04), while the smaller dAb penetrated the synapse most effectively (0.11±0.04) (Fig. 6b). Synapses formed by pNK cells (Fig. 6c) largely excluded mAb (0.03±0.01) while again, F(ab′)_2_ fragments were present within the synapse to some extent (0.06±0.04) but crucially, dAbs were able to penetrate the IS most efficiently (0.15±0.05; Fig. 6d). These data are consistent with the hypothesis that the IS excludes proteins in a size-dependent manner.

Low-density lipoprotein (LDL) can inhibit the action of perforin^27^ and therefore could potentially affect NK cell cytotoxicity if present in the synapse during secretion. To test whether or not LDL is present in the IS, pNK cells were co-incubated with 221 target cells in the presence of Alexa Fluor 488-labelled LDL and conjugates were imaged by confocal microscopy (Fig. 6c). LDL, shown to have diameter 21.4±1.3 nm (ref. 28), was largely excluded from the synapse (fluorescence intensity in the synapse relative to the solution 0.03±0.01; Fig. 6d)—to an extent similar to the exclusion of mAb. These data are consistent with the idea that a role of the synapse is to remove extracellular proteins in a size-dependent manner.

## Discussion

Here we set out to investigate whether or not the IS acts as a gasket. We found that the IS does not completely seal the synaptic cleft from the extracellular environment, rather the synapse forms a physical barrier that limits entry of extracellular molecules in a size-dependent manner. Larger molecules are cleared from the IS concurrent with the effector cell spreading response. For both NK cell and T-cell synapses, dextrans of hydrodynamic diameter 32 nm or more were largely excluded. Exclusion of molecules at the synapse requires an intact F-actin structure, evidenced by the fact that the addition of Lat A allowed 32-nm-sized dextran to enter the synapse. However, once the synapse has assembled active rearrangements of F-actin are not required to exclude larger-sized molecules, since Jasp does not affect the exclusion of large-sized dextran from the IS.

This exclusion and clearance of large molecules from the IS may have several important roles. First, the effector cell spreading response likely requires the clearance and exclusion of soluble molecules larger than the span of the synaptic receptor/ligand complexes. If large molecules were to be included within the synapse, adjacent to smaller receptor/ligand pairs, bending of the membrane would be a significant energetic penalty^29^. Receptor/ligand interactions at the T-cell or NK cell synapse span ~15–20 nm (refs 13, 30, 31), and the size of the NK cell synaptic cleft, measured by electron microscopy, was found to be between 15 and 25 nm with some areas of larger span^13^,^31^. This is consistent with molecules greater than this size being excluded.

Although the access of extracellular soluble proteins into the IS has not been explicitly tested before, segregation by molecular size has been studied mathematically in the context of the synaptic organization of cell-surface proteins^29^,^32^, which is important in the kinetic-segregation model of immune cell activation^33^. Previously, Köhler *et al*.^34^ demonstrated that longer receptor–ligand pairs segregated from shorter protein–protein interactions, and this can influence signal integration at the NK cell synapse. In a separate study by Alakoskela *et al*.^31^, quantum dots anchored to an NK cell membrane with a diameter of 23.4 nm were shown to accumulate at the periphery of the interface, while smaller quantum dots were included within the synapse. Altogether, these studies, coupled with the data presented here, demonstrate that size is a crucial factor in the assembly of the synapse. Thus, the clearance and exclusion of large molecules may be required to accommodate appropriate receptor/ligand interactions within the synapse at the lowest energy cost (that is, to minimize bending of the cell membrane).

Second, the structure of the synapse may serve to prevent the leakage of cytotoxic proteins that could detrimentally affect neighbouring cells, for example, perforin and granzymes that are secreted in complex with serglycin^35^, which would be larger than the size thresholds demonstrated here. However, some smaller molecules may be able to leak from the synapse. For example, Sanderson *et al*.^36^ have shown that interferon-gamma is able to leak from a cytotoxic T lymphocyte synapse. This cytokine is ~6 nm in its longest dimension^37^, smaller than the size threshold identified here.

A third consequence of the synapse acting as a size-dependent barrier may be that this protects synaptic secretions, such as perforin, from the action of serum proteins. Specifically here, we established that LDLs can be cleared and excluded from the IS. LDLs, for example, have been shown to inhibit the activity of perforin by binding to nascent hydrophobic domains used for membrane insertion^27^,^38^. These lipoproteins may serve to protect neighbouring cells from damage, should there be a loss of perforin from the synapse or from target cell itself. However, if these proteins were not cleared from the synapse, they could interfere with perforin activity, preventing the lysis of diseased cells.

The ligation of inhibitory receptors does not exclude larger-sized dextrans from the synapse, indicating that the exclusion of molecules is not solely due to protein–protein interactions connecting two cells together, but rather is a consequence of the activation and spreading of the effector cell. Thus, it may be important that this process clears large proteins from the synapse to prevent the interference with secreted proteins such as perforin.

An important outcome of the clearance and exclusion of larger molecules from the IS is that synaptic proteins will be protected from the action of relatively large therapeutic mAbs. There is anecdotal evidence that mAb-based therapies are unable to access target proteins secreted across the IS, such as perforin^9^. Here, we have established that small antibody-based proteins, such as dAbs, readily penetrate the IS. Thus, the development of small drugs, such as dAbs, to bind targets within the IS, for example, perforin, may provide a approach to treat or alleviate diseases, such as autoimmunity, where cell-mediated cytotoxicity contributes to pathology.

## Methods

### Cell isolation and culture

pNK cells were isolated by negative selection from healthy human donor peripheral blood (NK cell isolation kit; Miltenyi Biotec) as previously described^19^. The use of human blood was approved for Imperial College London by the Riverside Research ethics committee (05/Q0401/108) and for the University of Manchester by the University Research Ethics Committee (12295). Human blood from a leukocyte cone was poured in to a 250-ml flask and 50 ml RPMI 160 (equilibrated to room temperature) was then added (Sigma-Aldrich). In 50 ml tubes, 15 ml Ficoll-Paque (GE Healthcare) was added and an equal amount of the blood/RPMI 1640 mixture was added drop by drop. This was then centrifuged at 535 *g* for 40 min at 20 °C without a brake applied. Using a sterile plastic Pasteur pipette, the lymphocyte layer (second from top) was removed and placed into 50 ml tubes. The cells were then washed twice, adding RPMI 1640 to the lymphocytes to a final volume of 50 ml and centrifuged at 301 *g* for 10 min at room temperature. The supernatant was then decanted and cells were resuspended in 10 ml RPMI 1640, counted and 10^8^ cells were taken and RPMI 1640 added to a final volume of 5 ml. Tubes were then centrifuged at 301 *g* for 10 min at 4 °C and the supernatant was removed completely. The pellet was then resuspended in 400 μl MACS buffer (PBS, 2 mM EDTA (Sigma) and 0.5% BSA (Sigma); sterile filtered) and to this, 100 μl biotin–antibody cocktail (product code: 130-092-657; Miltenyi Biotec) was added, mixed and incubated for 5 min at 4 °C. After incubation, 300 μl MACS buffer and 200 μl anti-biotin microbeads (Miltenyi Biotec) were added, mixed and incubated for 10 min at 4 °C. Cells were then washed by centrifugation at 301*g* for 10 min at 4 °C and the supernatant was removed. Cells were then resuspended in 500 μl MACS buffer and placed in a prepared (rinsed with 3 ml MACS buffer, placed in a magnetic stand (Miltenyi Biotec)) LS column (product code: 130-042-401; Miltenyi Biotec) over 15 ml tubes. The column was then washed three times with 3 ml MACS buffer, allowing column to dry between each wash. The cells were then counted, pelleted by centrifugation at 301 *g*, 4 °C and resuspended in clone medium (described below) to a final cell density of 10^6^ cells per millilitre.

Freshly isolated cells were cultured in Dulbecco’s modified Eagle’s medium supplemented with 10% human serum, 2 mM L-glutamine, 1 mM sodium pyruvate, 1 mM penicillin/streptomycin and 1 mM non-essential amino acids (all obtained from Gibco, Life Technologies; referred to as clone medium), expanded with 150 U ml^−1^ human recombinant interleukin-2 (Roche) and then were rested for 6 days before use. Cell lines were cultured in RPMI 1640, 10% (vol/vol) fetal bovine serum (FBS), 2 mM L-glutamine, 50 U ml^−1^ penicillin, and 50 μg ml^−1^ streptomycin (Life Technologies), referred from hereon as R10. For imaging, cells were washed and resuspended in R10 prepared with phenol red-free RPMI 1640 (Life Technologies).

### Dextran

Dextran (labelled or unlabelled with dyes where indicated) of molecular weights 3, 10, 40, 70, 500 and 2,000 kDa were used (Molecular Probes; Life Technologies). Hydrodynamic radii of fluorescein-labelled dextran were previously measured by dynamic light scattering and were found to be 1.5, 2, 5, 6.5, 16 and 27 nm (refs 10, 11). Stock solutions of dextran were prepared in PBS and final concentrations of dextran used were (unless stated otherwise; for example, in experiments where concentrations were matched for comparison) 50 μM for the 3-, 4-, 10- and 13 nm dextrans, 5 μM for the 32-nm dextran and 2.5 μM for the 54-nm dextran.

### Antibodies and LDL

Goat anti-rabbit IgG antibodies labelled with Alexa Fluor 488 (A-11055, Life Technologies) and goat anti-rabbit IgG F(ab′)_2_ fragments labelled with Alexa Fluor 488 (A-11070, Life Technologies) were used at 3.25 and 4.5 μM, respectively. A domain antibody against glycoprotein VI (GlaxoSmithKline) was generated as described by Walker *et al*.^39^. The domain antibody was labelled with Texas Red using a Protein-X labelling kit (Life Technologies) according to the manufacturer’s instructions and was used at 3 μM. Acetylated LDLs labelled with Alexa Fluor 488 (Molecular Probes) were used at 1 μM.

### Live-cell imaging by confocal fluorescence microscopy

Live-cell imaging was performed using a commercially available laser scanning confocal microscope (TCS SP5 RS and TCS SP8 CW, Leica) equipped with × 63 and × 100 oil immersion objectives (numerical aperture (NA)=1.2 or 1.47, respectively). Nuclei of the cells were imaged using NucBlue (Hoechst 33342; *λ*_ex_=405, *λ*_em_=415–450; Molecular Probes). Dextran was imaged depending on its specific dye: fluorescein (*λ*_ex_=495, *λ*_em_=505–560), Texas Red (*λ*_ex_=595, *λ*_em_=605–660) and Alexa Fluor 647 (*λ*_ex_=647, *λ*_em_=657–700). Antibodies and LDL were imaged depending on their specific dye: Alexa Fluor 488 (*λ*_ex_=488, *λ*_em_=498–560) or Texas Red (*λ*_ex_=595, *λ*_em_=605–660). The theoretical spatial resolution in the *xy* axis and *z* axis were 280 and 1,086 nm for fluorescein, 337 and 1,306 nm for Texas Red and 360 and 1,418 nm for Alexa Fluor 647, respectively. Images were taken using an optical zoom between × 2 and × 4, giving a mean pixel size of 88±15 nm. All experiments were performed at 37 °C. Time-lapse images were acquired every 60–100 s over 15–20 min or every minute for 5 min. Images were analysed using ImageJ software (ImageJ; National Institutes of Health). Brightness and contrast were altered in some images to make the intensity within the synapse visible in the figures shown, but analyses were performed on raw images and data sets.

### Live-cell TIRF microscopy

Live-cell imaging was performed using a commercially available wide-field fluorescence inverted microscope (SR GSD 3D; Leica) in TIRF illumination mode with an HC PL APO × 160 oil immersion objective (NA=1.43) using an Andor iXon3 EMCCD camera. Dextran was imaged using either a 488- or 532-nm laser, depending on its specific dye: fluorescein (*λ*_ex_=488 nm) and Texas Red (*λ*_ex_=532 nm). Time-lapse images were acquired every 20 s for 5 min. Images were analysed using ImageJ software. Brightness and contrast were altered in some images to make the intensity within the synapse visible in the figures shown, but analyses were performed on raw images and data sets.

### T-cell synapse formation

To form T-cell synapses, 5 × 10^6^ Raji cells were incubated with 10 ng ml^−1^ enterotoxin E superantigen (Sigma-Aldrich) for 1 h at 37 °C. Cells were then washed three times with RPMI 1640 supplemented with 0.5% FBS and resuspended in 1 ml R10. Jurkat cells were mixed with Raji cells at a 1:1 ratio at a density of 10^5^ cells per millilitre.

### Live-cell confocal microscopy

Target cells were labelled with NucBlue (Molecular Probes; Life Technologies) according to the manufacturer’s instructions. YTS or pNK cells were co-incubated for times indicated at a 1:1 ratio at a density of 10^5^ cells per millilitre with dextran as indicated. Cells were plated in eight-well-chambered borosilicate coverglasses coated with 0.01% poly-L-lysine (Sigma-Aldrich) according to the manufacturer’s instructions, where indicated. Conjugates were imaged by confocal microscopy taking image stacks of 0.5 μm *z* axis step-size over 10 μm around the central plane of the synapse. Lat A and Jasp stocks were dissolved in DMSO. Where used, Lat A (Sigma-Aldrich) and Jasp (Life Technologies) were added to conjugates at final concentrations of 1 and 0.5 μM, respectively.

### Actin depolymerization assay

Lat A was added to 10^6^ cells at a final concentration of 1 μM and was incubated at 37 °C for 5 min. Cells were then washed and resuspended in 4% paraformaldehyde and incubated at room temperature for 20 min. Cells were then washed and resuspended in blocking/permeabilization buffer PBS containing 3% BSA (Sigma) and 0.02% Triton-X (Sigma) for 20 min at room temperature. Phalloidin conjugated to Alexa 647 (Molecular Probes) was then added (1:50), mixed by gentle shaking and incubated at room temperature for a further 30 min.

### Cell migration assay

To analyse cell migration ability and speed, a cell migration assay was used. First, 3 × 10^6^ pNK cells were stained with NucBlue according to the manufacturer’s instructions. Cells were then washed three times, resuspended in three separate 15 ml microcentrifuge tubes in 1 ml of clone medium each. Then, either 1 μl R10 medium, 1 μl DMSO or 1 μl Jasp (to a final concentration of 05 μM) was added to 10^6^ pNK cells resuspended in 1 ml clone medium and was incubated for 5 min at 37 °C 5% (vol/vol) CO_2_. R10 medium was then added to a final volume of 15 ml, and cells were washed by centrifugation at 1,300 r.p.m. for 5 min at room temperature and were resuspended in 1 ml clone medium. For imaging, 2 × 10^5^ cells were plated in eight-chambered borosilicate coverglasses coated with 1 μg ml^−1^ fibronectin and imaged by time-lapse confocal microscopy. Single-plane images were taken every 3 s for 10 min. Cell displacement was then measured using automated tracking software (Kalaimoscope; Transinsight).

### Flow cytometric analysis of cell viability

pNK, YTS and 221 cells were assessed for their viability after incubation with R10, 4% paraformaldehyde, DMSO, 1 μM Lat A or 0.5 μM Jasp for 5 min at 37 °C. Treated cells were washed and resuspended in flow buffer (0.5% fetal bovine serum/PBS) before staining with DAPI (Molecular Probes) according to the manufacturer’s instructions. Cells were then washed and were resuspended in flow buffer before analysis by flow cytometry (LSRFortessa, Becton Dickinson). For analysis, 10,000 intact events, as determined in forward scatter/side scatter plots were collected for each condition and data analysis was performed using FlowJo software version 9.7.6 (TreeStar).

### Time-lapse live-cell confocal microscopy

Where used, 221 target cells were labelled with NucBlue according to the manufacturer’s instructions. YTS and 221 target cells were co-incubated for times indicated at a 1:1 ratio at a density of 10^5^ cells per millilitre with dextran as indicated. Primary cells were used at a density of 10^5^ cell per millilitre with dextran as indicated. Cells were plated in eight-well-chambered borosilicate coverglasses coated with 0.01% poly-L-lysine (Sigma-Aldrich), 10 μg ml^−1^ fibronectin (both according to the manufacturer’s instructions) or 4 μg ml^−1^ MICA-Fc and 1 μg ml^−1^ ICAM1-Fc, as previously shown^19^, where indicated. Conjugates were imaged by confocal microscopy taking either image stacks of 0.5 μm *z* axis depth over 10 μm around the central plane of the synapse (conjugate imaging) or single-plane images (cell–slide interface) for the times indicated.

### Image and statistical analysis

Raw images were analysed using ImageJ software. To analyse the fluorescence intensity of dextran in cell–cell conjugates, a 50-pixel wide line was drawn across the conjugates, perpendicular to the synapse, placing the centre of the line at the point of cell–cell contact. The mean intensity along this line was used to produce a graph of dextran fluorescence intensity across the solution, cell bodies and synapse (Supplementary Figs 2,3 and 5). One-micron-wide boxes were drawn on the profile plots at the synapse and solution, and the area under the curves of fluorescence intensity was measured. The area (that is, the raw integrated intensity) at the synapse was then divided by the area at the solution to give the relative intensity of dextran within the synapse.

To analyse confocal imaging of pNK cells in contact with activating surfaces, the ImageJ plug-in ‘Time Series Analyzer, Version 4_2H’ (http://rsb.info.nih.gov/ij/plugins/time-series.html) was used. The mean intensity within a circular region at the point of cell–slide contact was measured and this was divided by the mean solution fluorescence intensity at the same time point to give a relative intensity of the synapse to the solution.

For TIRF microscopy, images were converted from 16- to 8 bit and an intensity threshold of 32–223 a.u. was applied. A look-up table, HiLo, was then used to identify pixels of the greyscale images with intensities below 32 a.u. (blue) and above 223 (red). To quantify the area of pixels below the threshold within the synapse interface, a threshold was first applied to the fluorescence image of the 32-nm dextran to identify and measure the area of the cell–slide interface. The raw fluorescence images were then converted into 8-bit images. To these images of 4 and 32 nm dextran, a threshold between 32 and 223 a.u. was applied. The image was then inverted (to measure pixels below threshold values) and a region of interest (ROI) was drawn around the interface at the cell–slide contact. Within the ROI, the area of pixels below the fluorescence threshold (blue) was then measured.

Data were analysed using GraphPad software (Prism). In Figs 1c,f,h, 2a,b,e,g, 3e, 4h, 5b,d,f and 6b,d; Supplementary Figs 1c,7h and 8b,d,e, a one-way analysis of variance with Tukey corrections was used. In Figs 1d,e and 3f,g; Supplementary Figs 1d, 4c,d and 7d, a two-tailed Student’s *t*-test with Welch’s corrections was used. Figure 4c,f shows a 95% confidence interval. All error bars show mean±s.d. Statistical significance is represented in graphs as *P<0.1, **P<0.01, ***P<0.001 and ****P<0.0001.

## Additional information

**How to cite this article**: Cartwright, A.N.R. *et al*. The immune synapse clears and excludes molecules above a size threshold. *Nat. Commun.* 5:5479 doi: 10.1038/ncomms6479 (2014).

## Supplementary Material

Supplementary Information Supplementary Figures 1-8.

Supplementary Movie 1 Dextran sized 4 nm is not cleared from the synapse during assembly. Primary NK cells were plated with Texas Red-labelled 4 nm dextran and fluorescein-labelled 32 nm dextran. Synapse formation with the slide was imaged by time-lapse total internal reflection fluorescence microscopy and images were taken every 20 seconds. Video shows the 4 nm dextran at the cell-slide interface during synapse formation. Fluorescence intensity threshold shows greyscale images with pixel intensities below 32 AU (blue) and above 223 AU (red). Scale bar; 10 μm. The movie corresponds to Figure 4g (upper row).

Supplementary Movie 2 Dextran sized 32 nm is cleared from the synapse during assembly. Movie shows time-lapse total internal reflection fluorescence microscopy as described for Supplementary Movie 1 showing the fluorescein-labelled 32 nm dextran. Scale bar; 10 μm. The movie corresponds to Figure 4g (lower row).
