# Supplementary material for: The immune synapse clears and excludes molecules above a size threshold
Source: Nat Commun. 2014 Nov 19;5:5479. doi: 10.1038/ncomms6479 (PMC4248232; doi:10.1038/ncomms6479)
Supplement: Supplementary Information — Supplementary Figures 1-8. [file ncomms6479-s1.pdf]

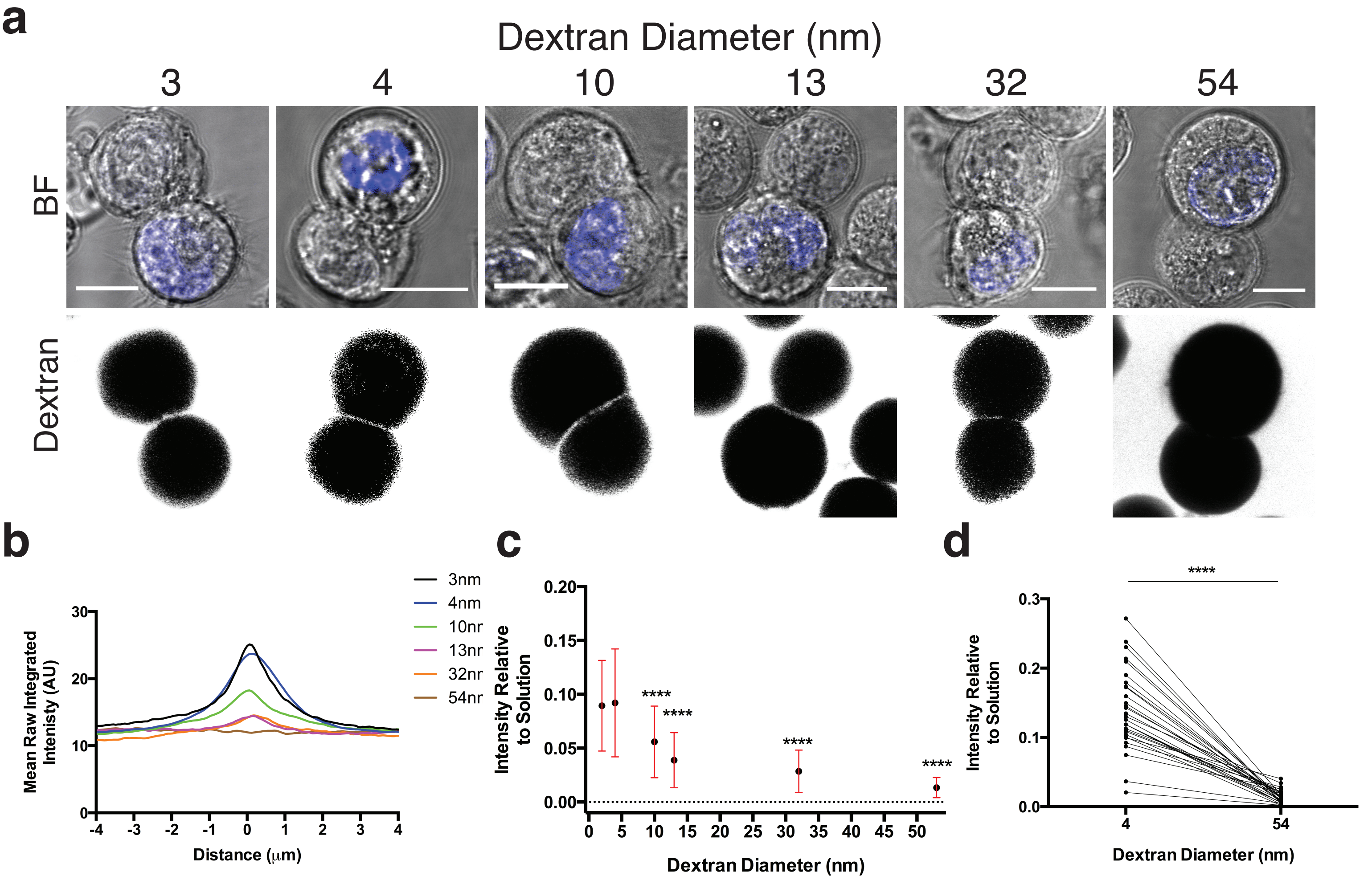

Supplementary Figure 1. The activating YTS cell immune synapse excludes molecules above a size threshold. (a) YTS cells were co-incubated with 221 target cells and fluorescently labelled dextrans of various sizes, as indicated. Panels show brightfield (BF) images overlaid with target cell nuclear stain (upper row) and the corresponding fluorescence image of dextran (lower row). Scale bar; 10 µm. (b) Graph shows the mean fluorescence intensity of various sized dextrans, as indicated, across the synapse. (c) Graph shows the relative intensity of dextran compared to the extracellular solution of each dextran size (3, 4, 10, 13, 3 and 54 nm dextran from left to right). Bars represent mean of all data points. n = 52, 55, 52, 51, 38 and 44 from three independent experiments. Data were analysed using a one-way ANOVA with Tukey corrections. ****p<0.0001. (d) YTS cells were co-incubated with 221 target cells, Texas Red-labelled 4 nm dextran and fluorescein-labelled 54 nm dextran. Graph shows the relative intensities of dextran within the synapse compared to the extracellular solution of the two differently sized dextrans within the synapse (connecting lines). n = 29 from three independent experiments. Data were analysed using a Student's t-test with Welch's corrections. ****p<0.0001.

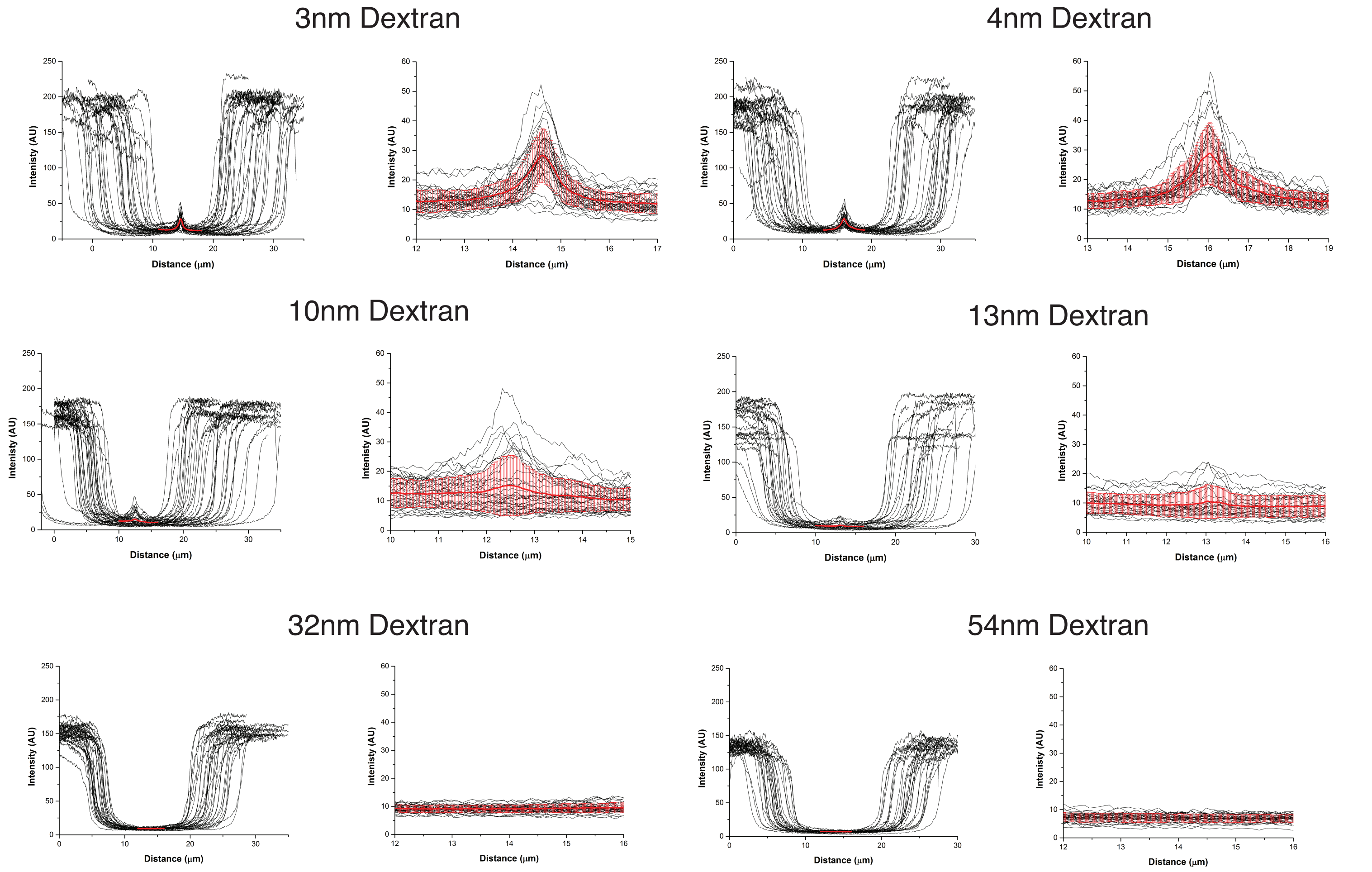

Supplementary Figure 2. Raw fluorescence intensity profiles of dextran across YTS cell-221 conjugates. Graphs show raw fluorescence intensity of dextran (sizes as indicated) across the extracellular solution, cell body and synapse (left panel) and a close up of the region across the synapse (right panel) of conjugates formed by pNK cells and 221 target cells. Red line and bars represents mean ± s.d. n = 38, 32, 33, 31, 38 and 35 for 3, 4, 10, 13, 32 and 54 nm dextran, respectively, from three independent experiments.

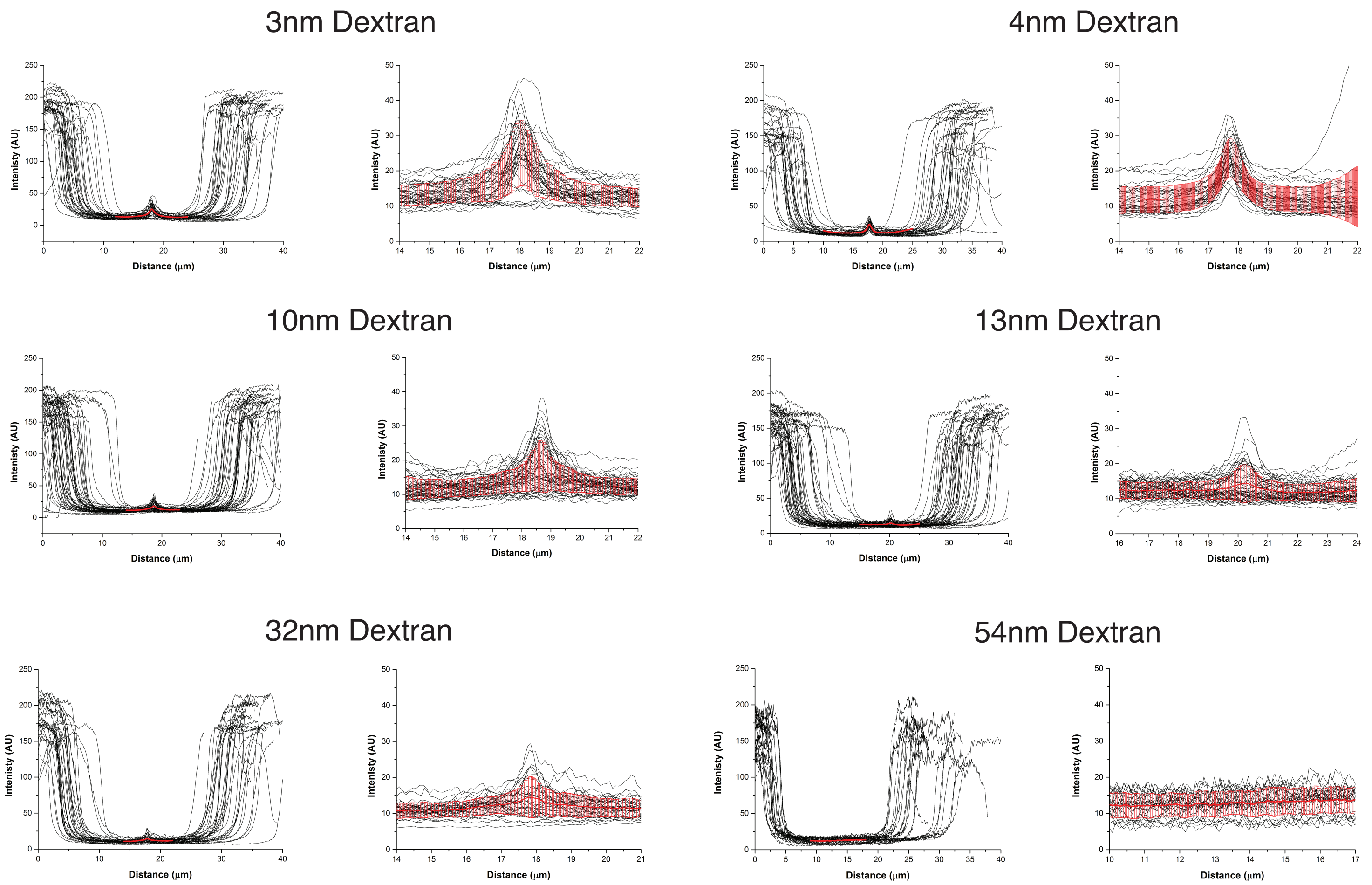

Supplementary Figure 3. Raw fluorescence intensity profiles of dextran across primary human NK cell-221 conjugates. Graphs show raw fluorescence intensity of dextran (sizes as indicated) across the extracellular solution, cell body and synapse (left panel) and a close up of the region across the synapse (right panel) of conjugates formed by primary human NK cells and 221 target cells. Red line and bars represents mean ± s.d. n = 49, 42, 47, 53, 39 and 39 for 3, 4,10, 13, 32 and 54 nm dextran, respectively, from three independent experiments.

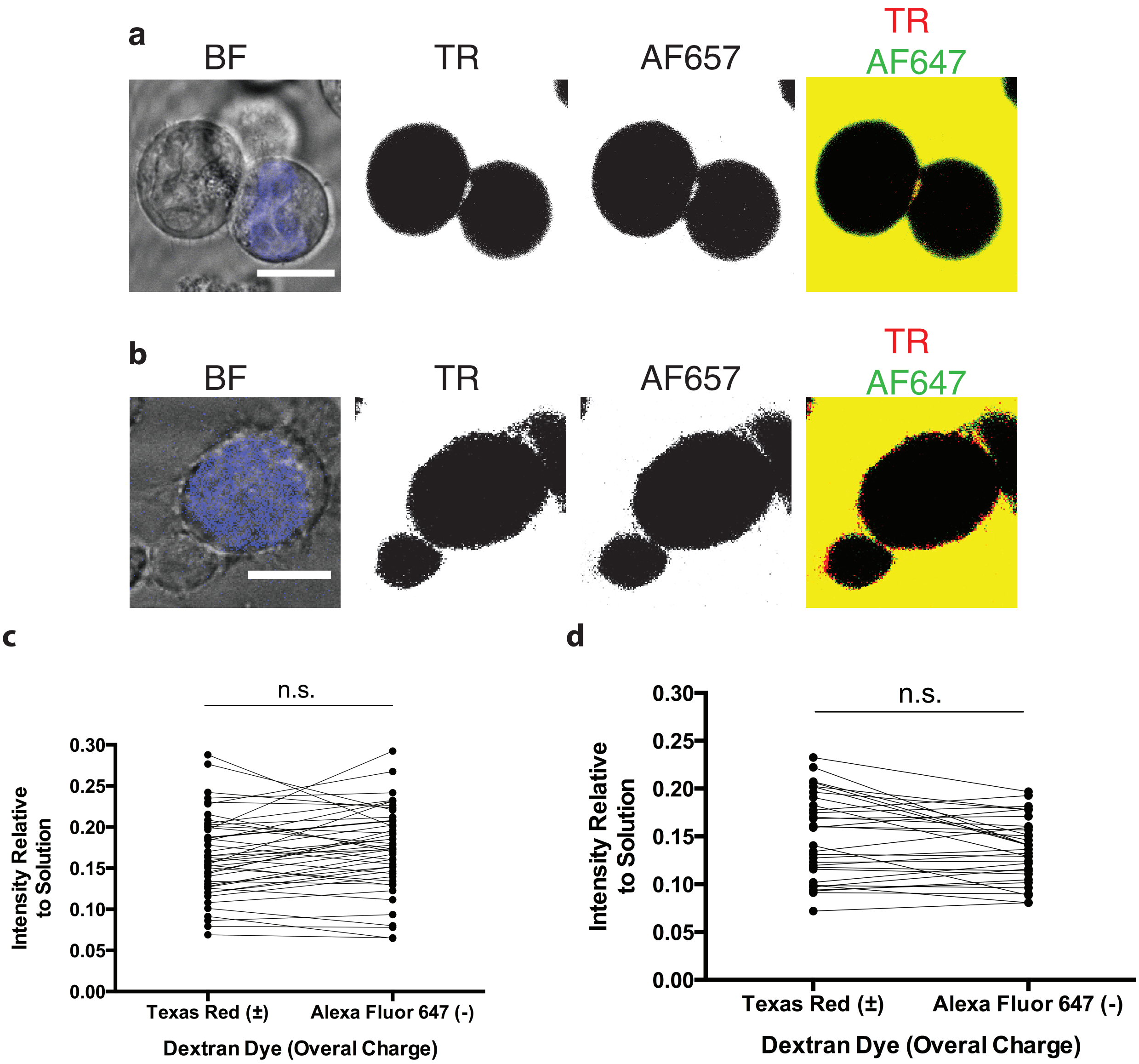

Supplementary Figure 4. Dextran charge does not affect its ability to enter the immune synapse. (a-b) Representative images of (a) YTS and (b) primary human NK cells in contact with 221 target cells co-incubated with differently labeled/charged dextrans as indicated. Panels show brightfield (BF) images overlaid with target cell nuclear stain and corresponding fluorescence images of fluorescent dextran labeled with Texas Red (TR) or Alexa Fluor 647 (AF647) and a merge of the fluorescent images. Scale bar, 10 µm. (c-d) Graphs show the mean relative fluorescence intensities of each dextran in the same synapse (indicated by connected lines) within synapses formed by (c) YTS or (d) primary NK cells with 221 target cells respectively. n = 45 for YTS cells and 32 for primary NK cells from three independent experiments. Data were analysed using a two-tailed Student's t-test with Welch's corrections n.s. = not significant.

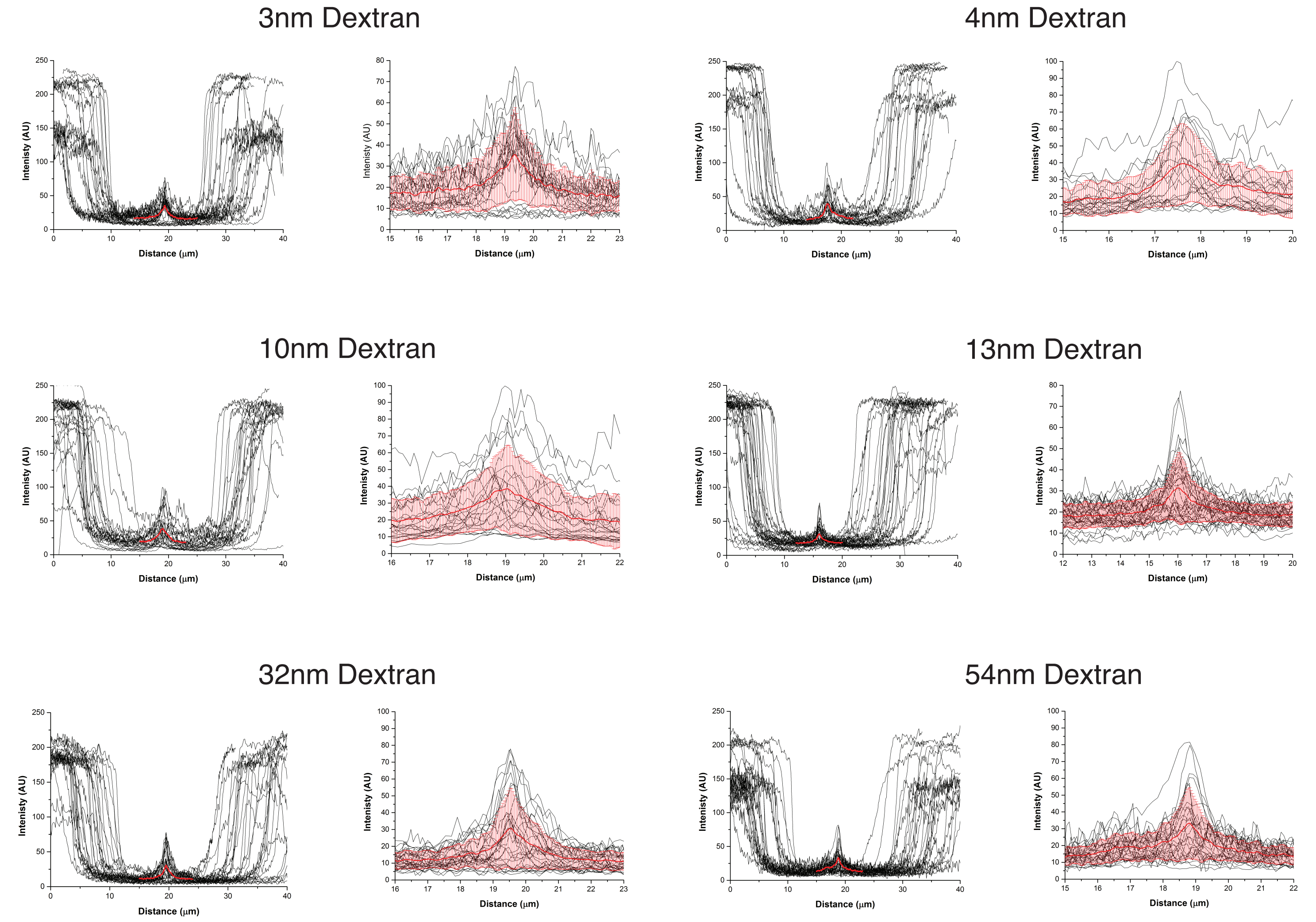

Supplementary Figure 5. Raw fluorescence intensity profiles of dextran across inhibitory NK cell immune synapses. Graphs show raw fluorescence intensity of dextran (sizes as indicated) across the extracellular solution, cell body and synapse (left panel) and a close up of the region across the synapse (right panel) of conjugates formed by YTS/KIR cells with 221/Cw6 target cells. Red line and bars represents mean ± s.d. n = 42, 45, 41, 45, 35 and 35 for 3, 4, 10, 13, 32 and 54 nm dextran respectively from three independent experiments.

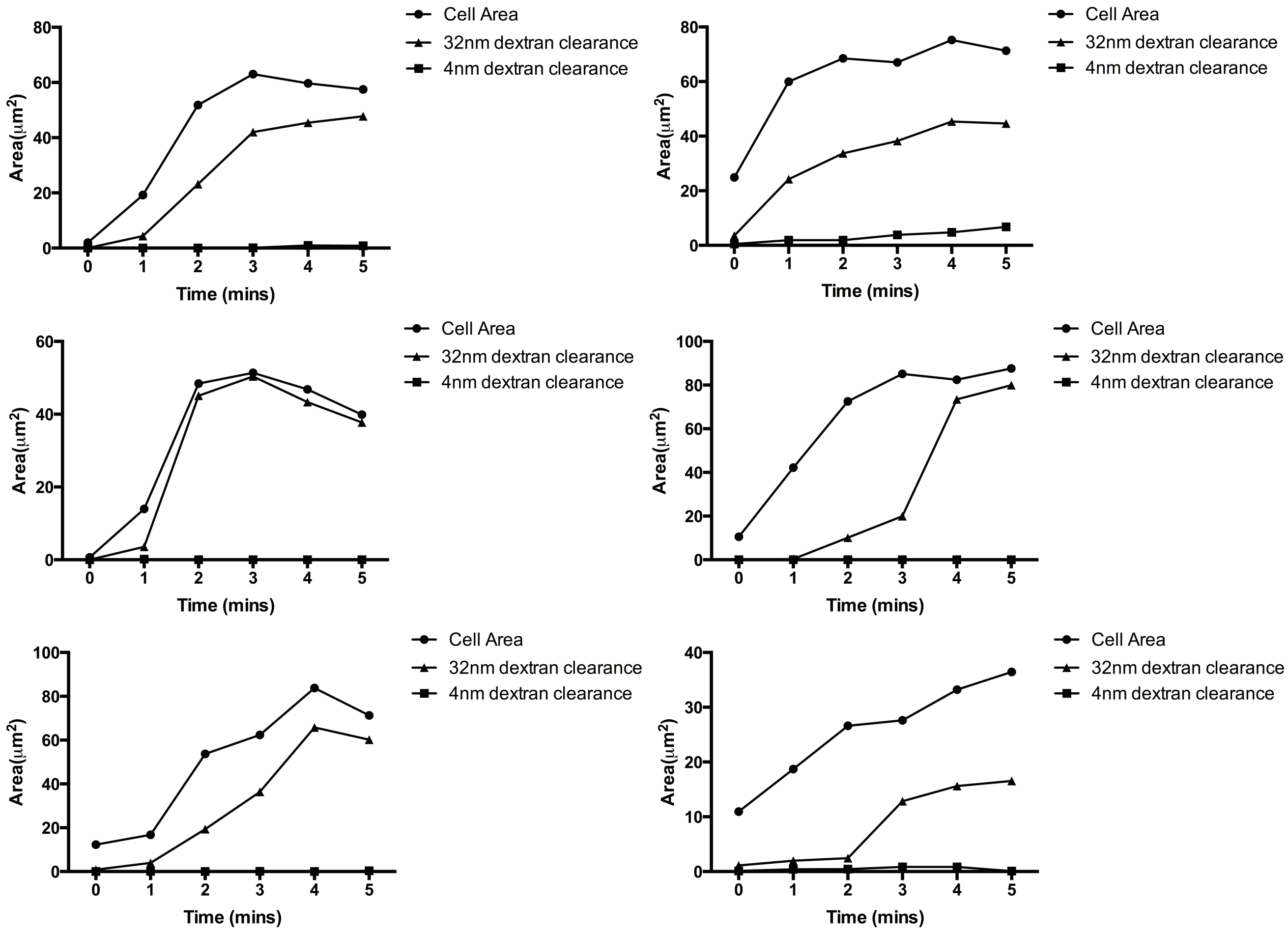

Supplementary Figure 6. Larger dextrans are cleared from the immune synapse. Primary human NK cells were plated on slides coated with MICA-Fc and ICAM1-Fc in the presence of Texas Red-labelled 4 nm dextran and fluorescein-labelled 32 nm dextran. Initial contact with the slide and synapse formation was imaged by total internal reflection fluorescence microscopy. Dextran clearance was quantified as intensity below a threshold of 32 AU. Graphs show the area of cell-slide contact and the area of pixels below 32 AU for 4 nm and 32 nm dextran at each time point indicated of each cell imaged (n = 6 from 5 independent experiments).

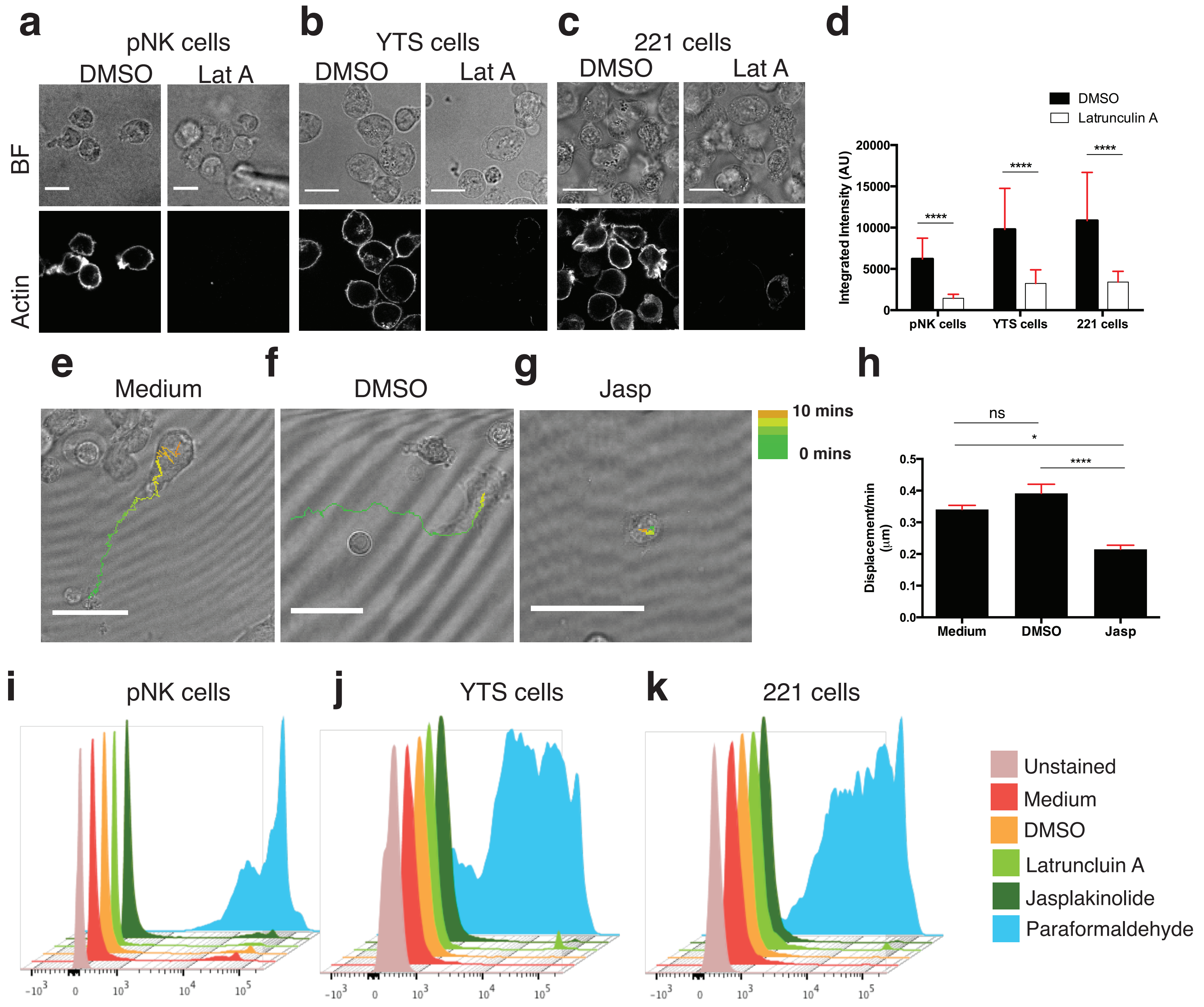

Supplementary Figure 7. Latrunculin A and Jasplakinolide affect actin structure and dynamics but not cell viability. (a–c) Primary human NK cells, YTS cells and 221 cells were incubated with a vehicle control, DMSO, or 1 µM Latrunculin A (Lat A) for 5 minutes. Cells were then washed, fixed, permeabilised and actin was stained using fluorescently labelled Phalloidin. Cells were then imaged by confocal microscopy. Panels show brightfield (BF) images (upper row) and the corresponding fluorescence image of Phalloidin-stained actin (lower row) of cells incubated with DMSO or Lat A, as indicated. Scale bar, 10 µm. (d) Actin integrated intensity was measured at the cell periphery for each cell type, as indicated, following incubation with DMSO (black bars) or Lat A (white bars). For pNK cells, n = 119 and 117 for DMSO and Lat A respectively. For YTS cells, n = 114 and 117 for DMSO and Lat A respectively. For 221 cells, n = 111 and 113 for DMSO and Lat A respectively. Data from 2 independent experiments. Data were analysed using a Student's t-test with Welch's corrections. $p<0.0001$. (e–g) Nuclear-stained pNK cells were incubated with medium, a vehicle control DMSO, or 0.5 µM Jasplakinolide for 5 minutes, washed, then plated on fibronectin-coated slides. Cell migration was imaged by time-lapse confocal microscopy for 10 minutes. Cell migration was then tracked using the nucleus as the cell centroid using automated tracking software. Migration is shown as a colour-coded track, corresponding to time scale indicated. Scale bar; 20 µm. (h) Graph shows the cell displacement/min following incubation with medium, DMSO or 0.5 µM Jasplakinolide, as indicated. Bars represent mean ± SEM. n = 34, 67 and 30 for medium, DMSO and Jasp respectively. Data from 2 independent experiments. (i–k) Primary human NK cells, YTS cells and 221 cells were incubated with medium and DMSO (negative controls), paraformaldehyde (positive control), 1 µM Lat A or 0.5µM Jasp for 5 minutes. Cells were then washed and incubated with DAPI to stain for cell death. Positive DAPI staining was then measured by flow cytometry. Data representative of 2 independent experiments.

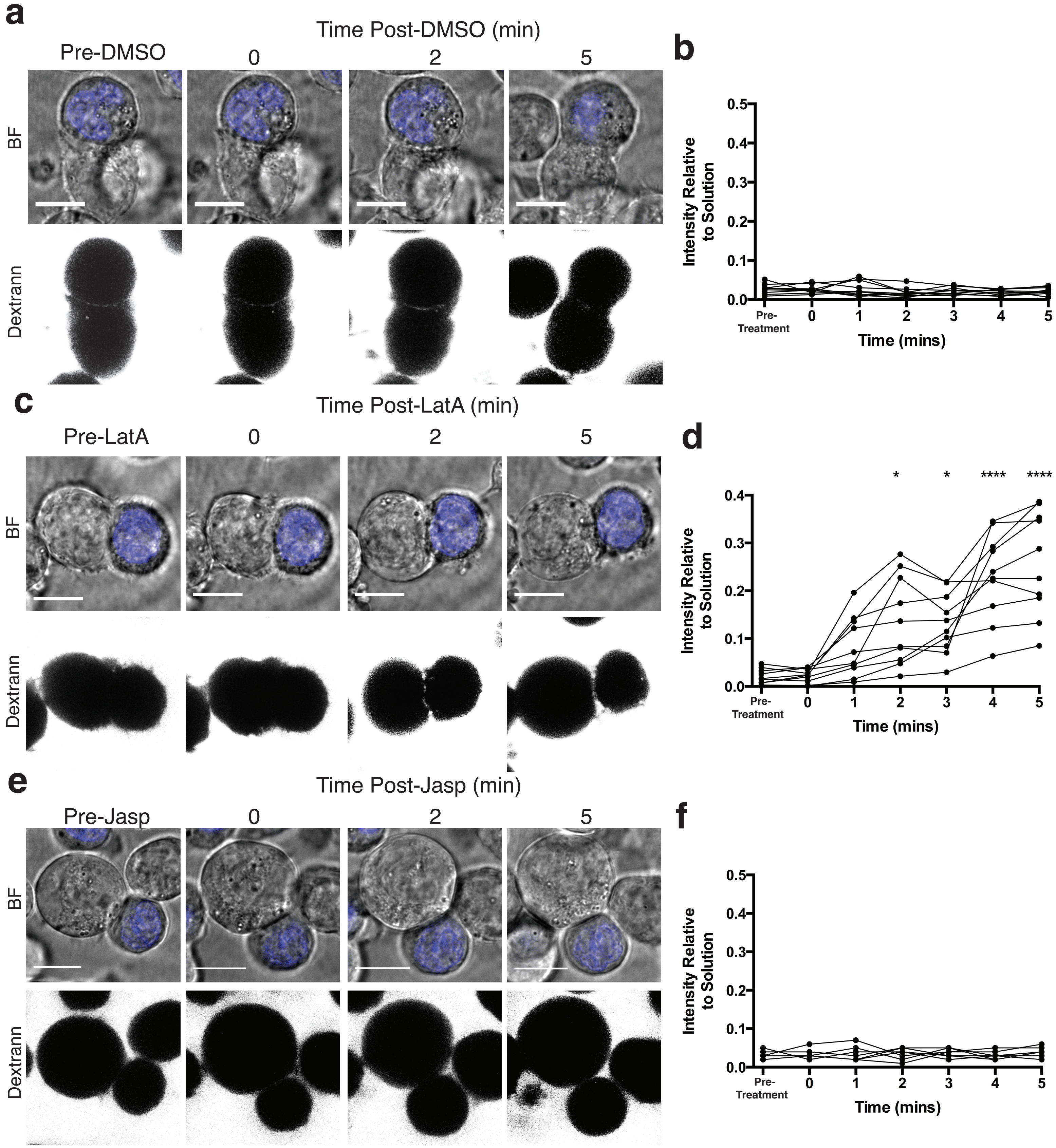

Supplementary Figure 8. The actin cytoskeleton is important for the exclusion of extracellular molecules from the synapse. YTS cells were co-incubated with 221 target cells and fluorescein-labelled 32 nm-sized dextran. Conjugates were imaged prior to and following the addition of DMSO, Latrunculin A or Jasplakinolide. Images show brightfield (BF) images overlaid with target cell nuclear staining and corresponding fluorescence images of 32 nm dextran prior to and following the addition of (a) DMSO, (c) Latrunculin A and (e) Jasplakinolide. Scale bar, 10µm. Graphs show the relative intensity of dextran within the same synapse at each time point (connecting lines) prior to and proceeding addition of (b) DMSO, (d) Latrunculin A and (f) Jasplakinolide, as indicated. Data are from 10, 10 and 7 conjugates for DMSO, Latrunculin A and Jasplakinolide, respectively. Data were analysed using a one-way ANOVA with Tukey corrections. $^{*}p<0.1$, $^{****}p<0.0001$.
